# Now you see it, Now you don’t: compulsive exercise in adolescents with an eating disorder

**DOI:** 10.1186/s40337-016-0129-8

**Published:** 2017-04-03

**Authors:** Johanna Levallius, Christina Collin, Andreas Birgegård

**Affiliations:** 1 Department of Clinical Neuroscience, Resource Center for Eating Disorders, Center for Psychiatry Research, Karolinska Institutet, SE-113 64 Stockholm, Sweden; 2 Resource Center for Eating Disorders, Stockholm Health Care Services, Stockholm County Council, SE-113 64 Stockholm, Sweden

**Keywords:** Eating disorder, Compulsive exercise, Compulsive exercise, Behavioral addiction, Adolescents, Denial

## Abstract

**Background:**

Compulsive exercise (CE) has been proposed as significant in the etiology, development and maintenance of eating disorders (EDs), resulting in more severe and enduring pathology. However, few studies have investigated CE longitudinally in adolescents with EDs. We aimed to test if adolescents show the same associations between CE and other clinical variables as previous research has found in adults.

**Methods:**

Three thousand one hundred sixteen girls and 139 boys from a clinical ED database were investigated regarding prevalence and frequency of CE and its relation to psychiatric symptoms, associated features and outcome. Denial of illness is common among adolescents and was therefore adjusted for.

**Results:**

Adjusted CE prevalence in girls was 44%, and CE was most prevalent in bulimia nervosa. As previously found in adults, those with CE scored significantly higher than non-CE on total ED severity, level of restriction and negative perfectionism. However, there were only minor differences between CE and non-CE patients on emotional distress, hyperactivity, suicidality and self-esteem. Among boys, adjusted CE prevalence was 38%, and CE boys scored significantly higher than non-CE on total ED severity. Initial CE did not influence 1-year outcome, although cessation of CE was associated with remission.

**Conclusions:**

CE is a common clinical feature in adolescents with EDs and cessation is associated with remission. When controlling for denial of illness, CE had less detrimental impact than predicted. We recommend controlling for denial in studies on ED adolescents and further exploration of classification and treatment implications of CE.

## Plain English Summary

The aims were to investigate how compulsive exercise (CE) related to eating disorder (ED) symptoms and outcome in adolescent girls and boys with EDs. We used a nationwide clinical ED database of 3116 girls and 139 boys. Forty-four percent of adolescents reported engaging in CE on average 3.9 times per week (*SD* = 2.6). Patients reporting CE had a slightly more severe clinical presentation. At 1 year, 55% had remitted from ED and prognosis was not affected by initial CE. However, cessation of CE increased the likelihood of remission from ED.

## Background

Physical exercise is regarded by both society and health care as a healthy way to diminish risk for, and detrimental effects of, several common diseases. However, from an eating disorders (EDs) perspective, exercise can be highly problematic. Compulsive exercise (CE) has been recognized as significant in the etiology, development, and maintenance of EDs [1–4], and is generally associated with more severe [5–7] and enduring ED pathology [8–10]. Both CE and EDs show detrimental effects on health-related quality of life, and interact to lessen quality of life further [11]. Moreover, rigorous exercise to control weight and/or shape has been linked to increased suicidality over and above other ED symptoms in both clinical and non-clinical adult samples [12]. EDs in themselves are serious illnesses and the co-occurrence of CE seems to exacerbate the condition, yet there is very limited research investigating this in adolescents. The current study therefore aimed to investigate the longitudinal relationship between different EDs and CE in the largest adolescent sample to date.

The terminology to describe problematic exercise has varied in the literature. The term compulsive exercise will be used here, as it is the most established term [13]. In an ED context, “CE describes a condition of weight and shape concern and a persistent continuation of exercise in order to (a) mitigate guilt/negative affect if not exercising, and (b) avoid perceived negative consequences of stopping” ([1], *p* 184). The two largest studies investigating CE among adults with EDs estimate a prevalence of 36–45%, varying depending on diagnostic subgroup and CE definition [6, 14]. The important factor seems to be presence or absence of CE rather than frequency, although CE among patients is more frequent than among healthy controls (9.9 vs 1.6 in 28 days) [15]. The few studies including adolescents found no difference compared to adults in prevalence or frequency of CE [4, 16], but a recent review found an average prevalence in studies of CE among adolescents with ED to be 52.6% [17]. In studies on adults, ED onset occurred at an earlier age in CE patients [6], who also showed more associated features such as perfectionism [6, 18], obsessive/compulsive behavior [19], and hyperactivity [16]. ED is consistently associated with low self-esteem, but whether patients with CE have even more impaired self-esteem is uncertain [20, 21]. Findings regarding whether CE is associated with depression and anxiety are also inconsistent [1, 17, 21].

Research findings detailed above pertain to females, as research on males with EDs and CE is scarce. Welch and colleagues [14] found comparable CE levels in men with ED as in women, despite lower ED symptomatology in men. In both sexes, exercise frequency has been linked to body dissatisfaction [22]. A large study on prediction of compulsive exercise in 12–14 year old normal boys and girls found the same three major predictors in both groups: drive for thinness, self-directed perfectionism and obsessive-compulsiveness [23].

Two aspects should be taken into account when examining exercise in EDs; one quantitative (frequency, duration, intensity), the other qualitative (compulsivity and rigidity) [24]. Several validated measures have been developed to capture various dimensions of these two aspects [13]. In this study, presence of CE was determined by a question in the Eating Disorder Examination Questionnaire (EDEQ), not originally designed to capture CE. However, studies have found strong correlations between this item and established CE measures [12, 13], supporting the use of EDEQ items as an approximation of CE.

Another factor to be taken into account is that minimizing or denying symptoms is common in adolescents with ED [25], possibly affecting the validity of symptom assessment. We therefore sought to explore this particular feature as a possible confounder, under the umbrella term “denial of illness”.

In summary, based on the studies reviewed above, our aims were to investigate how CE relates to ED diagnosis, symptoms, associated features and outcome in adolescents with ED, using a nationwide clinical database. Based on previous findings in adults, we expected similar CE prevalence in adolescents as in adults, and that CE would be associated with earlier ED onset, higher symptomatology, suicidality, negative perfectionism, hyperactivity, and a poorer prognosis. We did not expect effects of CE frequency or sex differences. No hypotheses were posited regarding CE and its relation to depression or self-esteem. In addition, we investigated the potential effect of denial of illness, a problematic feature particularly common in adolescents with ED.

## Methods

### Participants and procedure

Data came from the Stepwise database, a nationwide Swedish longitudinal clinical database of ED patients seeking treatment at specialist psychiatric treatment units [26]. All patients aged 13–17 registered in the database from 2005-03-07 (start of the database) through 2015-06-16 were extracted (*N* = 3982). Inclusion criteria were medical or self-referral to one of the participating treatment units, a diagnosed DSM-IV ED, and consent to research use of data. Three thousand two hundred and fifty-five patients fulfilled inclusion criteria, and 95.7% were girls. Diagnoses were anorexia nervosa (AN), bulimia nervosa (BN) and ED not otherwise specified (EDNOS). Patients received treatment of various modalities (e.g., medical, psychological, pedagogical, nutritional, social, physical), length, and intensity (e.g., residential, day and/or out-patient treatment).

During the time of data collection, between 60 and 90% of specialist units participated, and coverage ratio within units was high (>90%). Stepwise assessment, including interviews and self-report questionnaires, was performed within the first three visits (for inpatients, within the first week of care), and self-report instruments had satisfactory psychometric properties. *Age at onset* was defined as the patient’s retrospective report of symptom debut. Remission was defined as not fulfilling any ED diagnosis according to the semi-structured EDs interview described below. Ethical approval was granted by the regional review board (No. 2009/1298-31/1, EPN Stockholm).

### Measures

*The Structured Eating Disorder Interview* (SEDI) is a semi-structured computerized interview with 20–30 questions, used to assess DSM-IV diagnostic criteria, with number of questions automatically varying depending on presenting symptoms [27]. BMI cutoffs defining underweight for AN were adjusted for age and sex, based on Swedish norms. Preliminary validation against the EDE interview has shown a concordance of 81% regarding specific ED diagnosis (including EDNOS and BED) and Kendall’s tau-b of 0.69 (*p* < .001) [27].

*The Eating Disorder Examination Questionnaire* (EDEQ, adolescent version) [28] estimated ED symptoms. EDEQ consists of 36 items, 23 of them rated on a seven degree scale. Scores on items are averaged into four subscales; restraint, eating concern, weight concern, and shape concern, averaged into a global score. Thirteen items cover presence and frequency of binge eating, self-induced purging, and compulsive exercise. Patients were divided into CE or non-compulsive exercisers (non-CE) by the dichotomous “*Have you exercised hard to control weight or shape?*”. This was followed by a CE-frequency question, pertaining to the last 14 days. The EQEQ for adolescents has failed to reproduce the original four factor structure [29]. However, for the purpose of this study we only included frequency items, the restraint subscale and the global score, which have shown satisfactory psychometric properties [29].

*Mini International Neuropsychiatric Interview for Children and Adolescents* (MINI-Kid) is a psychometrically sound semi-structured interview of Axis-I disorders, performed by clinicians consisting of several modules. Here we used data from the A module, which assesses caseness of depression, and the B module, which assesses suicidality, the latter both as presence/absence and a three-stage level of risk [30].

*The Strengths and Difficulties Questionnaire* (SDQ) measures self-rated behavioral and psychological problems among children and adolescents in 25 items rated on a 3° scale covering *emotional distress*, *behavioral problems*, *hyperactivity/inattention*, and *problems with peers*. It also includes a fifth positive scale, *prosocial behavior*. Based on the theoretical aims, the subscales emotional distress and hyperactivity/inattention were used. The SDQ has acceptable internal consistency, test-retest stability, and content validity [31].

*The Structural Analysis of Social Behaviour* (SASB) measures self-image in 36 items, with statements rated on a 0–100 (10-point increment) scale [32]. The SASB model is a circumplex organized around two orthogonal dimensions: the horizontal Affiliation axis, ranging from self-love to self-hate/self-hostility, and the vertical Autonomy axis, ranging from self-control to spontaneity/letting go. Matching our aims, we selected two variables: the *Affiliation vector* (computed as a weighted summary score for items pertaining to the Affiliation axis [32] that has been shown to approximate self-esteem [33]), and *Self-criticism*, involving self-blame over mistakes, self-accusation, and negative comparisons to others, which approximates negative perfectionism.

### Statistical analysis

Analyses were carried out using SPSS Version 22. Independent two-tailed *t*-tests were used for comparisons between CE and non-CE. Cohen’s *d* effect size was assessed according to small (≥.20), medium (≥.50), and large (≥.80) conventions. Chi-square test was used to test for differences in proportion of CE patients by diagnostic subgroup, depression and suicide risk. Pearson correlations were computed to investigate the relationship between the different variables and CE frequency, while linear regression was used to test if CE frequency predicted severity at 1 year. Logistic regression was used to test if initial presence of CE could predict remission. Alpha was set at *p* ≤ .001 for the full subsample of girls due to the large *n*, and *p* ≤ .05 for boys as well as for girls when divided into diagnostic ED subcategories. To adjust for outliers, a maximum CE frequency of 30 in the last 2 weeks was set (<0.1% of ratings). *Denial of illness* was defined as scoring below age-, sex-, and diagnosis-adjusted clinical cutoff on the EDEQ global score, using Clinical Significance (CS) calculations as described in Ekeroth and Birgegård [34].

## Results

To first address denial of illness, we explored the proportion scoring below EDEQ clinical range. 25% of adolescents with EDs rated below clinical cut-off; this was most common among AN patients (31% of AN patients denied illness). Patients below cut-off were termed ‘deniers’ and those above ‘clinical’. Deniers generally rated lower on all psychopathology, including CE, where prevalence was 12% versus 44% for the clinical group. Further analyses were therefore performed in two ways: for all patients and for clinical patients only.

### Girls

CE proportion for all girls was 36%, and mean frequency was 3.9 times/week (*SD* = 2.62). For all girls and the clinical group respectively, CE prevalence differed significantly by diagnosis (χ^2^ (2, 3116) = 88.813, *p* < .001, and χ^2^ (2, 2345) = 49.120, *p* < .001), and was most prevalent in BN (Table 1). Excluding deniers increased overall prevalence to 44%, with the largest increases in AN and EDNOS.

**Table 1:** Group characteristics by diagnosis in girls

All girls								
	AN	(*SD*)	BN	(*SD*)	EDNOS	(*SD*)	Total	(*SD*)
*n*	1112		329		1675		3116	
Age	15.1	(1.35)	16.2	(1.04)	15.4	(1.33)	15.4	(1.34)
Body mass index	15.8	(1.42)	22.0	(3.91)	19.2	(2.97)	18.3	(3.35)
EDEQ global	3.1	(1.60)	4.2	(1.08)	3.4	(1.53)	3.39	(1.55)
CE (%)	26		50		40		36	
CE frequency	7.4	(6.5)	6.3	(3.9)	7.2	(5.2)	7.1	(5.4)
Clinical group								
*n*	772		306		1267		2345	
Age	15.1	(1.35)	16.2	(1.06)	15.4	(1.31)	15.4	(1.33)
Body mass index	15.9	(1.41)	22.0	(3.79)	19.5	(2.95)	18.6	(3.40)
EDEQ global	4.0	(1.01)	4.4	(0.79)	4.2	(0.86)	4.13	(0.91)
CE (%)	34		53		48		44	
CE frequency	8.5	(6.4)	6.7	(3.7)	8.0	(5.1)	7.9	(5.3)

CE frequency reported for last 14 days

Note: *AN* anorexia nervosa, *BN* bulimia nervosa, *EDNOS* eating disorder not otherwise specified, *BMI* body mass index, *EDEQ* eating disorder examination questionnaire, *CE* compulsive exercise

Independent *t-*tests showed that CE patients differed significantly from non-CE patients on several measures: global EDEQ, restraint, negative perfectionism, emotional distress, hyperactivity, and self-esteem (Table 2). However, exclusion of deniers reduced effect sizes considerably. Fifty percent were diagnosed with depression in the clinical group (41% for all girls), with chi-square test finding no significant difference in prevalence of depression between CE and non-CE patients (χ^2^ (1, 2170) = 1.083, *p* = .30). Contrary to hypothesis, CE was not associated with suicide risk (χ^2^ (1, 2063) = 0.018, *p* = .89). CE frequency correlated significantly but weakly with EDEQ global (*r* = .176), restraint (*r* = .161), binge frequency (*r* = .248), negative perfectionism (*r* = .100), and hyperactivity (*r* = .124).

**Table 2:** Group comparisons of symptomatology in CE vs. non-CE female patients

	All girls				Clinical group			
	CE	Non-CE	*t*	*d*	CE	Non-CE	*t*	*d*
Age at onset	13.8 (1.7)	13.7 (1.9)	0.03	-	13.7 (1.7)	13.7 (1.9)	0.56	-
EDEQ								
Global	4.1 (1.1)	3.0 (1.6)	23.61*	0.87	4.4 (0.9)	4.0 (0.9)	10.85*	0.46
Restraint	4.1 (1.4)	2.7 (1.9)	22.82*	0.86	4.3 (1.3)	3.7 (1.4)	10.60*	0.44
Binge (*M*)	7.6 (10.1)	7.7 (10.8)	0.20	-	7.8 (10.4)	8.5 (11.2)	1.11	-
Purge (*M*)	7.6 (9.1)	8.3 (9.7)	1.01	-	7.8 (9.2)	8.6 (10.0)	1.21	-
SASB								
Negative perfectionism	58.7 (23.7)	44.7 (25.9)	15.34*	0.56	61.1 (22.4)	55.7 (21.9)	5.94*	0.25
Self-esteem	−13.9 (35.2)	6.1 (41.2)	14.26*	0.56	−18.0 (32.5)	−12.2 (32.9)	4.28*	0.18
SDQ								
Emotional distress	6.2 (2.3)	5.3 (2.6)	10.42*	0.41	6.5 (2.1)	6.3 (2.1)	1.91	-
Hyperactivity	4.9 (2.3)	4.1 (2.4)	9.68*	0.36	5.0 (3.3)	4.6 (2.3)	3.88*	0.16

Note: *CE* compulsive exercise, *EDEQ* eating disorder examination questionnaire, *SASB* structural analysis of social behavior, *SDQ* strengths and difficulties questionnaire

* = *p* < 0.001

The characteristics of CE patients might differ depending on ED diagnosis. The pairwise comparisons above were therefore repeated for the three diagnostic subgroups, excluding deniers. As depicted in Table 3, CE patients were highly similar to each other on all variables investigated, regardless of ED diagnosis. When looking at pairs within each diagnosis, differences emerged mainly for AN patients, where CE patients scored higher than non-CE on ED symptomatology, negative perfectionism, and self-esteem. Chi-square tests showed no significant difference in prevalence of depression between pairs (AN χ^2^ (1, 723) = 1.212, *p* = .15, BN χ^2^ (1, 285) = 0.732, *p* = .23 EDNOS χ^2^ (1, 1162) = 0.754, *p* = .21), yet suicide risk was significantly more prevalent in AN with CE (AN χ^2^ (1, 678) = 6.471, *p* = .01, BN χ^2^ (1, 274) = 0.000, *p* = 0.55 EDNOS χ^2^ (1, 1111) = 2.734, *p* = .06).

**Table 3:** Symptomatology in clinical girls with CE vs. non-CE by ED diagnosis

	AN				BN				EDNOS			
	CE	Non-CE	*t*	*d*	CE	Non-CE	*t*	*d*	CE	Non-CE	*t*	*d*
Onset	13.4 (1.8)	13.6 (1.8)	0.98	-	13.7 (1.8)	13.1 (2.9)	1.24	-	13.7 (1.7)	13.7 (2.1)	0.44	-
EDEQ												
Global	4.3 (1.0)	3.8 (1.0)	6.56*	0.50	4.5 (0.8)	4.3 (0.82)	2.32	-	4.3 (0.8)	4.0 (0.9)	7.28*	0.41
Restraint	4.4 (1.3)	3.7 (1.4)	6.92*	0.52	4.1 (1.2)	3.8 (1.43)	1.97	-	4.3 (1.2)	3.7 (1.4)	7.85*	0.44
SASB												
Negative perfectionism	63.4 (22.3)	52.9 (23.4)	6.00*	0.45	63.2 (21.1)	61.0 (21.5)	0.89	-	59.6 (22.6)	56.7 (20.4)	2.44	-
Self-esteem	−20.0 (34.3)	−8.5 (34.8)	4.39*	0.33	−20.5 (29.2)	−19.8 (32.1)	0.20	-	−16.4 (32.5)	−13.3 (31.3)	1.74	-
SDQ												
Emotional distress	6.5 (2.2)	6.0 (2.2)	2.89	-	6.5 (2.1)	6.5 (2.1)	0.16	-	6.5 (2.1)	6.4 (2.1)	0.12	-
Hyperactivity	5.0 (2.3)	4.4 (2.2)	3.47	-	5.1 (2.2)	5.4 (2.4)	0.92	-	5.0 (2.4)	4.6 (2.3)	2.53	-

Note: See Table 2 for explanation of abbreviations

* = *p* < 0.001

One-year follow-up data was available for 52% of the sample. There was a significant overall decrease in global EDEQ score (*M*_*diff*_ = 1.90, *t* = 32.38, *p* < .001) and the majority (55%) had also remitted from ED. CE patients were hypothesized to have a poorer remission rate and this was disconfirmed, both for all girls and the clinical group χ^2^ (1, 1059) = 2.450, *p* = .12 and χ^2^ (1, 825) = 0.627, *p* = .43. CE frequency at baseline did not predict either remission (logistic regression χ^2^ (1, 399) = 1.77, *p* = .18) or symptom improvement as measured by EDEQ (linear regression *F*[1, 381] = .081, *p* = .78). At follow-up, there was a certain degree of cross-over, meaning most patients ceased to utilize CE as compensatory behavior (69% of CE patients) whereas others began (17% of non-CE) (Table 4). Those that ceased with CE had a greater likelihood of remission compared to those who continued (χ^2^ (1, 381) = 22.33, *p* < .001), see Table 4.

**Table 4:** Remission rate of clinical girls by CE status at baseline and follow-up (*n* = 779)

Distribution of patients	CE at baseline	CE at 1 year	EDEQ baseline	EDEQ 1 year	Remission rate
45%	No	No	3.95 (0.88)	1.74 (1.47)	56%
32%	Yes	No	4.22 (0.89)	1.72 (1.50)	59%
14%	Yes	Yes	4.43 (0.78)	3.61 (1.55)	29%
9%	No	Yes	4.15 (0.85)	3.47 (1.42)	39%

Note: *CE* compulsive exercise, *EDEQ* eating disorder examination questionnaire

### Boys

Twenty-nine percent of boys reported CE (38% in the clinical group), with a mean frequency of 4.1 times/week (*SD* = 2.83), see Table 5. As with CE girls, CE boys had significantly higher global EDEQ and restraint score, and reported more negative perfectionism, emotional distress, and hyperactivity (Table 6). Performing analyses again after excluding deniers (35 CE and 57 non-CE remaining), effect sizes were reduced even more than for girls. The only significant difference remaining was that CE boys had higher Global EDEQ than non-CE boys. Twenty-eight percent of boys had depression, with higher prevalence in the CE group (41%) (χ^2^ (1, 130) = 4.947, *p* = .03), but CE was not associated with suicide risk (χ^2^ (1, 119) = 0.584, *p* = .45). CE frequency did not correlate significantly with any variables in Table 6 (*r* range = −.155–.257). Sample size did not permit analyses by ED subgroup.

**Table 5:** Group characteristics by diagnosis in boys

	AN	(*SD*)	EDNOS	(*SD*)	Total	(*SD*)
All boys						
*n*	40		96		139	
Age	15.0	(1.35)	15.2	(1.33)	15.1	(1.33)
Body mass index	16.1	(1.42)	19.8	(2.97)	18.8	(3.68)
EDEQ global	2.1	(1.60)	2.1	(1.53)	2.2	(1.62)
CE (%)	25		29		29	
CE Frequency	6.5	(3.78)	8.4	(6.44)	7.9	(5.81)
Clinical group						
*n*	24		65		92	
Age	15.0	(1.16)	15.1	(1.43)	15.1	(1.34)
Body mass index	16.2	(1.07)	20.4	(4.07)	19.4	(4.08)
EDEQ global	3.3	(1.23)	2.9	(1.19)	3.1	(1.24)
CE (%)	38		39		38	
CE Frequency	5.6	(3.36)	7.7	(5.05)	7.1	(4.48)

Note: See Table 1 for list of abbreviations. Boys with bulimia nervosa omitted (*n* = 3)

**Table 6:** Group comparisons of symptomatology in CE vs. non-CE male patients

	All boys				Clinical group			
	CE	Non-CE	*t*	*d*	CE	Non-CE	*t*	*d*
Age at onset	14.0 (1.45)	13.7 (1.75)	0.77	-	13.9 (1.50)	13.7 (1.68)	0.63	-
EDEQ								
Global	3.0 (1.37)	1.8 (1.59)	4.13*	0.71	3.4 (1.04)	2.9 (1.31)	2.07*	0.46
Restraint	3.0 (1.64)	1.7 (1.84)	3.97*	0.76	3.4 (1.38)	2.7 (1.72)	1.94	-
Binge (*M*)	3.9 (2.71)	7.0 (7.99)	−1.76	-	4.2 (2.72)	6.3 (4.47)	−1.53	-
SASB								
Negative perfectionism	38.6 (23.39)	25.6 (24.06)	2.92*	0.55	42.5 (22.71)	36.2 (24.93)	1.23	-
Self-esteem	25.1 (35.59)	34.6 (40.30)	−1.31	-	19.0 (34.32)	15.7 (38.54)	0.43	-
SDQ								
Emotional distress	4.6 (2.68)	3.4 (2.49)	2.65*	0.48	5.1 (2.44)	4.3 (2.44)	1.68	-
Hyperactivity	4.6 (2.20)	3.5 (2.66)	2.49*	0.45	4.9 (2.21)	4.1 (2.61)	1.44	-

See Table 1 for explanation of abbreviations

Note. *CE* compulsive exercise

* = *p* < .05

At 1-year follow-up, 58% of boys had remitted, and initial CE status did not affect prognosis (χ^2^ (1, 45) = .046, *p* = 0.55) for boys either. Overall, there was a significant decrease in global EDEQ score (*M*_*diff*_ = 1.37, *p* < .001), where an independent *t-*test showed that CE patients again did not differ from non-CE patients (*t* = 0.214, *p* = .83) at 1-year.

## Discussion

This is the largest longitudinal study to date investigating presence of CE in adolescents with EDs. We found comparable CE prevalence among adolescents (44%) as previous research has reported in adults [6, 14], and comparable also to previous average estimates in ED adolescents [17]. Among girls, CE was more common in BN and EDNOS than in AN, a pattern also found in a previous representative Swedish study on adults [14]. After controlling for denial of illness, there was less difference between CE and non-CE patients on symptomatology and associated features than findings on adults would suggest [1, 16–21]. The study also included a sizeable number of boys, presenting the same general pattern as girls; however not all comparisons could be carried out for boys due to sample size. Overall, and contrary to studies on adults [8–10], CE adolescents did not have a worse prognosis in comparison to non-CE adolescents, provided they ceased with CE. At 1 year follow-up, two-thirds of adolescents reporting CE had ceased doing so, and remission rate was similar for them as for non-CE adolescents (59% vs. 56%). Adolescents still reporting CE at follow-up had lower remission rate (29%), as did those who had begun with CE during treatment (39%). The general finding in the current study of weaker associations than expected between CE and ED features might be due to a preponderance of inpatients and AN patients in studies (e.g., [8–10, 13, 16, 19–21]), where findings might not be generalizable to ED patients.

CE girls and boys alike rated higher ED symptomatology, in line with numerous previous studies on female adults [5–7]. At the subgroup level, CE was associated with higher negative perfectionism in AN girls, due to non-CE girls with AN actually rating a lesser degree of negative perfectionism than BN and EDNOS. CE was also associated with higher frequency of suicidality in AN girls; paradoxically not accompanied by elevated emotional distress or depression. Considering an elevated suicide risk in AN patients [35] in general, this merits further investigation. CE was associated with elevated ED symptom levels in AN and EDNOS, but not in BN. CE was furthermore not associated with depression, self-esteem (except in clinical AN) or hyperactivity, and it is not clear if CE itself may have buffered these factors, by mitigating negative affect [36], shame [37], and excess energy levels [16], or if CE is unrelated to them. Lastly, in line with our hypothesis, CE frequency had little impact on investigated associations, even though frequency ranged from twice a day to once biweekly.

### Theoretical and treatment implications

The variety of definitions and assessment methods for CE poses challenges in comparing studies. One other large scale study on a representative sample of adults has used EDEQ items [14], corroborating our estimates for adolescents. However, estimating CE by two self-rated EDEQ items has limitations. Despite being the most established ED measure, it does not capture all aspects of CE. Evidence however suggests that the EDEQ does capture the global concept fairly well, having a correlation of .64 with the Compulsive Exercise Test [13] and .57 with the Obligatory Exercise Questionnaire [12]. Thus, the EDEQ may profitably be used as a preliminary indication of CE, to then be assessed in greater detail with measures specifically designed to do so. Furthermore, given the high prevalence of CE in EDs, and its possible detrimental impact, perhaps CE has earned DSM criterion status in ED subcategories other than BN, where it is already included.

Some patients remitting from ED continued with CE, or began with CE during follow-up. CE is thus a common and problematic feature within EDs but not restricted solely to them, in line with other research findings [11, 23, 38]. The most prominent feature of CE pathology within EDs, put forth by Boyd and colleagues [39], is withdrawal symptoms if not exercising, in the form of guilt and feeling annoyed or agitated. Emotional withdrawal symptoms of this kind may be exacerbated in ED specifically due to strict self-imposed rules for weight/shape control. This is suggested also by ED research on the association between CE and negative perfectionism [6, 18] and drive for thinness [5, 8], and also consistent with our findings. Negative reinforcement is also a driving force behind addictions [40]. As with other behaviors under the umbrella term of *behavioral addiction*, where CE has been suggested to be appropriately placed [38, 40], the activity itself is not detrimental (cf. sex, work, internet use, shopping), so abstaining is neither possible nor desirable. Instructing the patient to refrain from exercising altogether may alleviate CE, but would be equivalent to ‘throwing the baby out with the bathwater’; as the many physiological and psychological benefits of exercise would be lost [41]. In the acute phase of stabilization, restricting energy expenditure might be necessary. Over time however, clinicians may do well in helping the patient both to eat and to exercise for the right reasons, in a balanced and flexible manner, while also teaching adaptive emotion regulation strategies. A recent study has shown positive effects of supervised exercise on emotional state in adolescent inpatients with AN [42], although it remains to be seen if such interventions are beneficial for CE or the ED itself.

An important aspect to hold in mind as well is that care should be taken not to overpathologize exercise or other behaviors [43]. There might be a compulsive element to a behavior such as exercising but unless there is stability over time and clear functional impairment it does not classify as addiction [43]. Problematic over-involvement in exercise has been shown to be fairly transient in a 5-year cohort study, even when untreated [44]. Clinical guidelines on treatment of CE are however lacking, as are intervention studies specifically directed at CE. This would be a welcome next step of investigation.

### Denial of illness

Denial falsely inflated differences between CE and non-CE patients. Despite receiving an ED diagnosis, 25% of adolescents rated below clinical cut-off and self-report of CE was substantially lower among them (12%), as was reporting of bingeing, purging and associated features. In this study, it was not possible to determine the underlying cause(s) behind denial, but the phenomenon clearly complicated assessment. Denial as a potential threat to validity should therefore be taken into account in studies on adolescents with EDs.

### Strengths and limitations

As mentioned, a specific measure of CE was lacking and frequency estimates were based on recall, entailing inherent instability. Also, we did not measure duration of CE engagement, threatening the validity of the compulsivity aspect. A specific measure for negative perfectionism was also lacking. However, item content and theoretical underpinnings of SASB Self-criticism are consistent with the negative perfectionism construct, and results were consistent with previous research. The present study has several strengths as well: the large sample size, the longitudinal design, inclusion of understudied groups such as boys and EDNOS, inclusion of all levels of severity and controlling for denial. Patients were included nationwide, with high coverage ratio, ensuring high ecological validity. Well validated and structured methods for diagnostics and self-rating were used. An attrition rate of 48% is potentially a threat to generalizability, yet attrition analyses found no significant differences, in concordance with another study on the database [45]. Patients in the current study received treatment of various modalities (e.g., medical, psychological, pedagogical, nutritional, social, physical), length and intensity (e.g., inpatient, day-patient, out-patient). Although outcome was similar in CE and non-CE patients, it is not clear if they benefit equally from particular interventions, and this was not investigated. Recalling the instability of problematic behaviors such as exercise [44], assessment of the role of CE in the symptom pattern of the individual is warranted, as is attention to the possibility that patients may switch between CE and other compensatory/addictive behaviors [46]. Clinically, different addictive or compulsive behaviors may be present interchangeably if the underlying function (emotion regulation) is not addressed.

## Conclusions

About four in ten adolescents with ED report engaging in CE. Patients with CE have a more severe clinical presentation, albeit not affecting 1-year prognosis. Cessation of CE during follow-up was associated with remission. Systematic assessment of CE would aid in estimating its impact on ED, in tailoring treatment, and in evaluating outcome. Denial of illness is a common feature in adolescents with EDs and as such a potential confounder in research.

## Abbreviations

CE: Compulsive exercise
ED: Eating disorder
EDEQ: Eating disorder examination questionnaire
MINI-Kid: Mini International neuropsychiatric interview for children and adolescents
SASB: Structural analysis of social behavior
SDQ: Strengths and difficulties questionnaire
SEDI: Structured eating disorder interview
